# Pulmonary tumor embolism secondary to soft tissue and bone sarcomas: a case report and literature review

**DOI:** 10.1186/s12957-017-1223-3

**Published:** 2017-08-30

**Authors:** Nicholas Latchana, Vincent C. Daniel, Robert W. Gould, Raphael E. Pollock

**Affiliations:** 1 Department of Surgical Oncology, University of Toronto, Toronto, ON Canada; 2 Grant Medical Center, Columbus, OH USA; 3 Department of Anesthesiology, The Ohio State University Wexner Medical Center, Columbus, OH USA; 4 Department of Surgery, Division of Surgical Oncology, The Ohio State University Wexner Medical Center- The Arthur G. James Comprehensive Cancer Center and Richard J. Solove Research Institute Center, 410 W. 10th Ave, N924 Doan Hall, Columbus, OH 43210 USA

**Keywords:** Sarcoma, Tumor embolism, Cor pulmonale

## Abstract

**Background:**

Tumor embolisms (TE) are an underappreciated source of pulmonary embolisms in sarcoma. Most evidence in the literature is limited to case reports and none have described the presence of TE secondary to myxofibrosarcoma. We report the first case of myxofibrosarcoma TE and perform a review of the literature for TE secondary to bone and soft tissue sarcomas (STS).

**Case presentation:**

A 36-year-old female presented with debilitating pain of the right upper extremity secondary to a recurrent soft tissue sarcoma. She had distant metastasis to the lung. An MRI revealed a 25-cm shoulder mass involving the proximal arm muscles with encasement of the axillary artery, vein, and brachial plexus. A palliative forequarter amputation was performed and tumor thrombus was evident within the axillary artery and vein. Postoperatively, she developed an acute onset of dyspnea and hypoxia. A computed tomography scan revealed a pulmonary saddle embolism. A bilateral lower extremity venous duplex was negative. She became hemodynamically unstable despite resuscitation and was placed on vasopressor support. A transthoracic echocardiogram revealed elevated pulmonary artery pressure, tricuspid regurgitation, right heart dilation, and reduced right heart systolic function consistent with acute cor pulmonale. The patient did not want to pursue a median sternotomy with pulmonary artery embolectomy and expired from cardiopulmonary arrest within 24 h of the operation. The final pathology revealed a 25 × 16 × 13 cm high-grade myxofibrosarcoma with invasion into the bone, skin, and neurovascular bundle as well as evidence of tumor thrombus.

**Conclusion:**

TE is a rare but deadly cause of pulmonary embolism in sarcoma. A high index of suspicion is necessary in individuals who present with respiratory-related symptoms, especially dyspnea. Diagnostic confirmation with a computed tomography scan of the chest and echocardiogram should be rapid. Unlike venous thromboembolism, pulmonary embolectomy remains the preferred therapeutic approach.

**Electronic supplementary material:**

The online version of this article (doi:10.1186/s12957-017-1223-3) contains supplementary material, which is available to authorized users.

## Background

Sarcomas are solid tumors originating from mesenchymal tissue that account for less than 1% of all adult malignancies [1]. This diverse group of neoplasias is classified into soft tissue sarcoma (STS) and bone sarcomas based upon the putative derivation of the primary tumor [2, 3]. Notably, the 5-year survival of STS and bone sarcoma is 66.4 and 52.9%, respectively [4].

Pulmonary embolism (PE) is a major cause of sarcoma-related death. An investigation of 252 individuals with STS and bone sarcomas revealed 1.2% had PE and 0.4% had a fatal PE [5]. Deaths are attributed, in part, by the progression to cor pulmonale which is defined by the presence of right ventricular dilation from pulmonary hypertension in either the presence or absence of right ventricular hypertrophy [6]. Non-venous thromboembolism (VTE) causes of PE must be considered in patients with cancer and include tumor, bacterial, mycotic, amniotic, and fat embolisms [7, 8]. The distinction between PE subtypes is important as the prognosis and management may differ [9].

Tumor embolism (TE) has been reported in 0.3–26% of patients with a solid malignancy at autopsy and is associated with a mortality rate of 8% [7, 8, 10, 11]. TE occurs most commonly from mucin-producing tumors of the breast, lung, colon, and stomach but can also occur from sarcoma [12]. An evaluation of 1457 cadavers in patients previously diagnosed with neoplasias revealed 10% had a TE [13]. Of these, sarcomas accounted for 13% of TEs [13]. Evidence pertaining to TE in the setting of metastatic sarcoma has been limited. A small case series involving six patients with bone sarcoma has been previously published but no sizeable case series are present for TE in STS or bone sarcoma [14]. Notably, TE secondary to myxofibrosarcoma has not been previously described. Articles pertaining to primary sarcoma of the pulmonary artery as well as direct extension of peripheral sarcoma to the pulmonary artery without embolization have been described elsewhere and were excluded from this analysis [15, 16]. Herein, we present the first reported case of TE secondary to myxofibrosarcoma followed by a review of the literature regarding metastatic TE in STS and bone sarcoma with emphasis on clinical presentation, pathophysiology, diagnostic measures, and therapeutic strategies.

## Case presentation

A 36-year-old female presented with a recurrent right upper extremity STS. Two years prior to initial presentation at our facility, she was diagnosed with an 11-cm right axillary, intermediate-grade pleomorphic spindle cell neoplasm. She underwent neoadjuvant chemoradiation therapy followed by a resection of the tumor. Microscopic margins were positive following the surgical resection and adjuvant chemotherapy was initiated. She developed a local recurrence 3 months after resection which ultimately progressed to invasion of the brachial plexus with paralysis of the right upper extremity as well as a pathologic humeral fracture. She was placed on low molecular weight heparin after developing a right subclavian vein thrombosis. Additionally, she developed distant metastatic disease in the left upper lobe of the lung. She perused several experimental modalities that were unsuccessful at reducing the tumor burden.

**Fig. 1:**
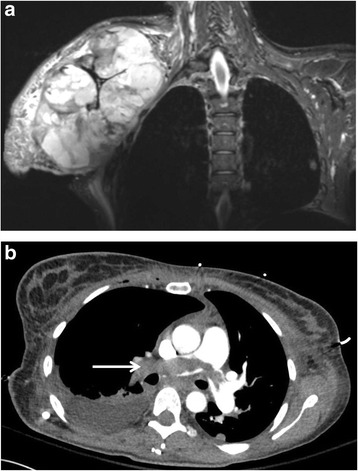
Primary tumor and pulmonary embolism. **a** Coronal chest MRI of a 36-year-old female with a recurrent right upper extremity myxofibrosarcoma. **b** Chest computed tomography of pulmonary artery saddle embolism (*arrow*)

She presented to our institution 2 years after the initial diagnosis with debilitating pain of the right upper extremity. An MRI revealed a 25-cm shoulder mass involving the proximal arm muscles with encasement of the axillary artery, vein, and brachial plexus (Fig. 1a). After a multidisciplinary discussion, a palliative forequarter amputation was performed. Tumor thrombus was evident within the axillary artery and vein during ligation.

Ten hours after completion of the operation, she developed an acute onset of dyspnea with a resultant increase in her supplemental oxygen requirement. A chest x-ray was obtained which revealed bilateral atelectasis. Suspicion of a pulmonary embolism remained high and confirmatory testing was performed with a computed tomography (CT) chest (Fig. 1b). A pulmonary saddle embolism was present on the CT scan and she was started on an intravenous heparin infusion. A bilateral lower extremity venous duplex study did not reveal a deep vein thrombosis. Her blood pressure subsequently decreased, and she was unresponsive to crystalloid intravenous administration; therefore, she was placed on vasopressor support. A transthoracic echocardiogram (TTE) was performed and revealed an elevated pulmonary artery pressure (49 mmHg), tricuspid regurgitation, right heart dilation, and reduced right heart systolic function consistent with acute cor pulmonale. The patient declined a median sternotomy with pulmonary artery embolectomy. She went into cardiopulmonary arrest and expired within 24 h of the operation. An autopsy was declined but TE was presumed to be the source of PE due to the presence of tumor invasion into the vein.

The final pathology of the primary tumor revealed a 25 × 16 × 13 cm high-grade myxofibrosarcoma. The tumor invaded the bone, skin, and neurovascular bundle and contained tumor thrombus.

## Results

**Fig. 2:**
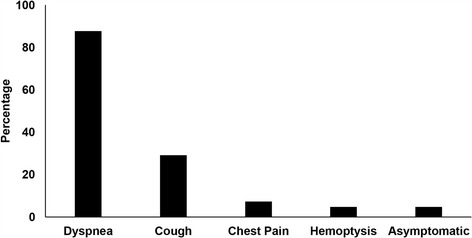
Clinical symptoms in patients with tumor embolism. The presenting symptoms of 45 patients with tumor embolism secondary to soft tissue and bone sarcomas are reported as a percentage of total patients

Including our case report, 40 publications that met criteria were included in this series. Together, these studies included 45 patients with TE secondary to STS (*n* = 14) or bone sarcoma (*n* = 31) (Additional file 1). The average age of patients was 36.6 years old (range 9–73) with 23 females (52.3%) and 21 males (47.7%) (1 not specified). Dyspnea was the most common clinical symptom (87.8%) followed by cough (29.0%), chest pain (7.0%), and hemoptysis (5.0%) while, 4.8% of patients were asymptomatic (Fig. 2). In studies with available data, most patients had an acute (0–2 weeks) onset of symptoms (*n* = 16). Seven patients had a subacute (2–8 weeks) onset of symptoms, and one patient had a chronic (> 8 weeks) duration of symptoms before seeking medical care.

**Fig. 3:**
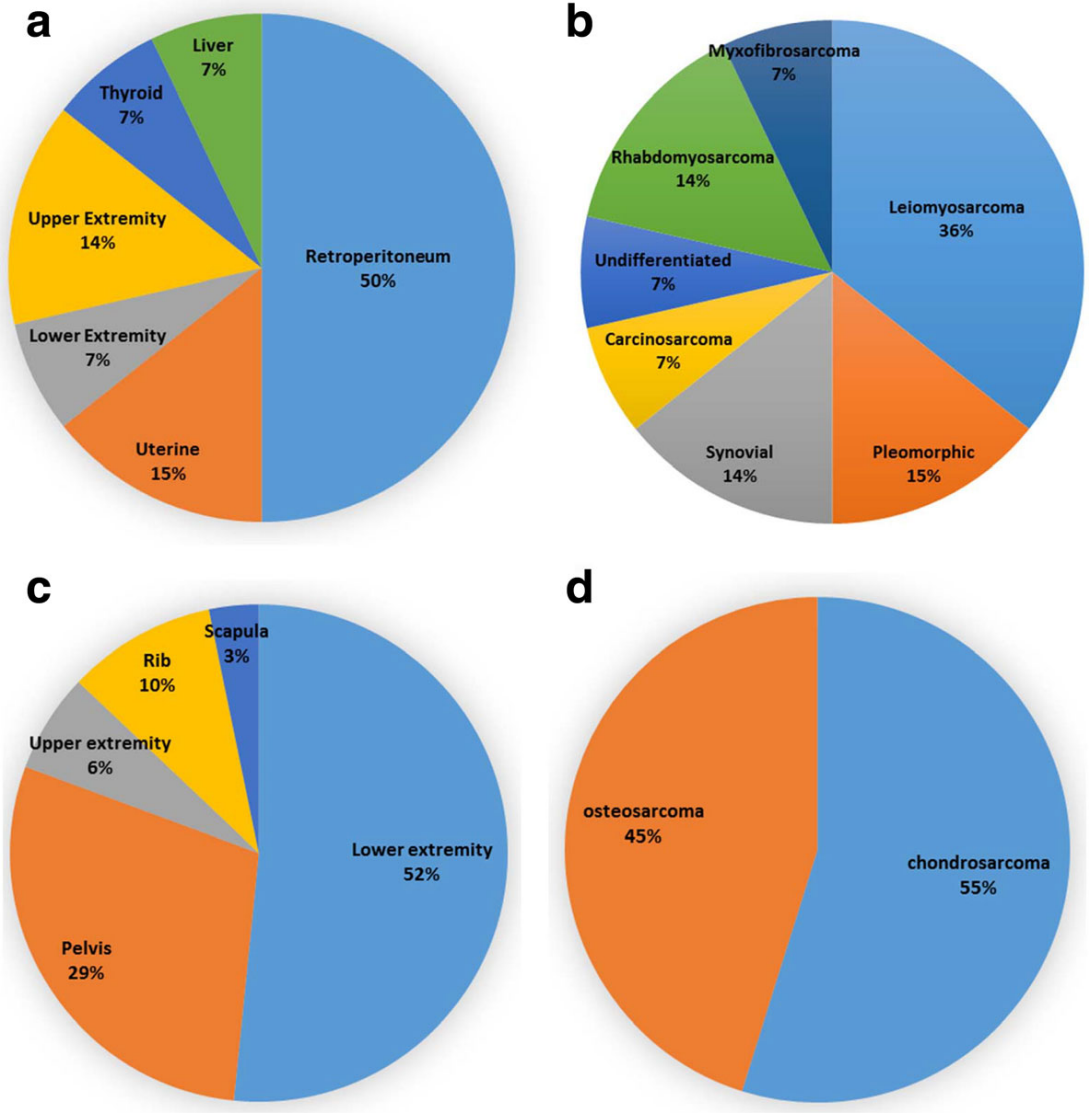
Site of primary tumor and histology of bone and soft tissue sarcoma. The location of the primary tumor and histology of patients with tumor embolism secondary to soft tissue sarcoma *n* = 14 (**a**–**b**) and bone sarcoma *n* = 31 (**c**–**d**), respectively, is reported as a percentage of total patients for each subtype

This study includes the first case of myxofibrosarcoma associated with TE. Primary STS were most commonly located in the retroperitoneum (*n* = 7) (Fig. 3a). The most common STS with TE in this series was leiomyosarcoma (*n* = 5) followed by pleomorphic, rhabdomyosarcoma, and synovial sarcoma (*n* = 2 each) (Fig. 3b). Conversely, the lower extremity (*n* = 16) and pelvis (*n* = 9) were the most common sources of TE in the setting of bone sarcoma (Fig. 3c). Chrondrosarcoma (*n* = 17) was the most common bone sarcoma while the remainder of cases were comprised of osteosarcoma (*n* = 14) (Fig 3d).

**Fig. 4:**
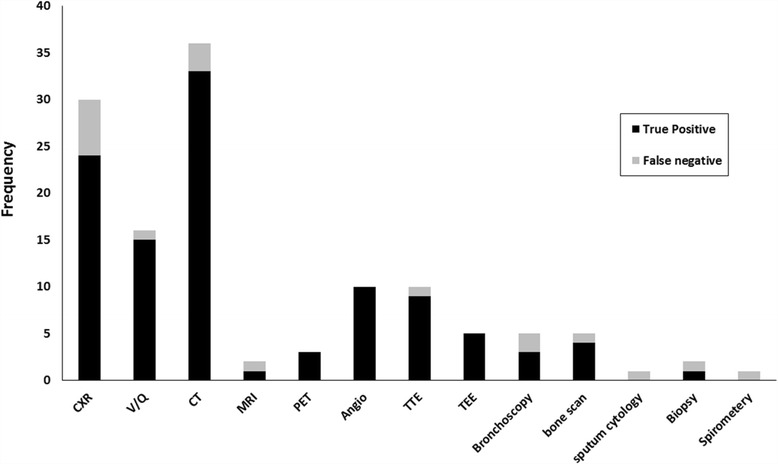
Diagnostic modalities in tumor embolism evaluation. The frequency of diagnostic tests used during the evaluation of 45 patients with tumor embolism secondary to soft tissue and bone sarcoma is represented. For each diagnostic modality, true positives (*black*) and false negatives (*gray*) are shown

The diagnosis of TE was not established until postmortem evaluation in 22.2% of cases. A variety of diagnostic modalities were used. CT scan (*n* = 36) was the most common modality utilized followed by chest roentgenography (*n* = 30), lung perfusion scans (*n* = 16), angiography (*n* = 10), and transthoracic echocardiography (*n* = 10) (Fig. 4). Chest radiography had the greatest number of false negative studies (*n* = 6). Additionally, evidence of cor pulmonale was found in 19 cases.

**Fig. 5:**
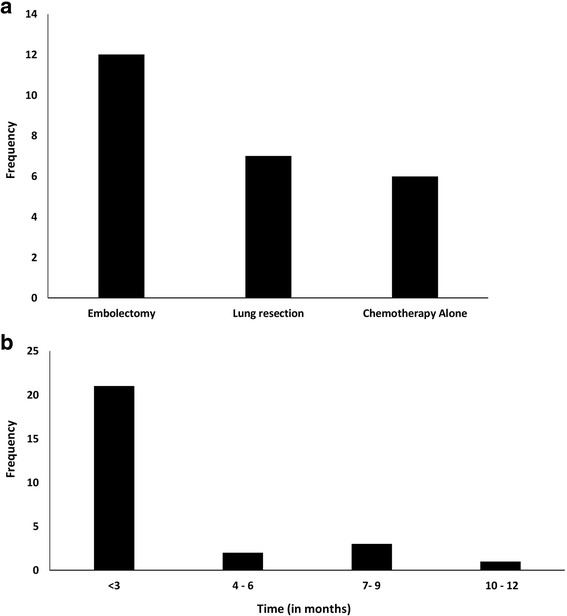
Management and outcome of tumor embolism. **a** Frequency of therapeutic modalities is shown for 25 patients who received definitive management of tumor embolism secondary to soft tissue and bone sarcomas followed by **b** duration of survival (in months)

Venous duplex of extremity/inferior vena cava (IVC) was performed in 11 cases and diagnostic of a deep vein thrombosis in 5/11 cases. Direct tumor venous invasion at the primary tumor site was noted in eight cases (Additional file 2). Anticoagulation was administered in 21 cases and 4 patients also received thrombolysis. Treatment of TE included embolectomy (*n* = 12), lung resection (*n* = 7), and chemotherapy alone (*n* = 6) (Fig. 5a). Death occurred in 64.3% (*n* = 27) of cases and most commonly ensued within 3 months (*n* = 21) (Fig. 5b). Notably, 16 of 18 (88.9%) of patients with cor pulmonale died (1 case with cor pulmonale did not have survival data). All patients that did not receive a definitive intervention for a diagnosed PE died (*n* = 7). Death occurred in three of four cases where an IVC filter (*n* = 3) or IVC plication (*n* = 1) was performed as the sole management of PE (follow-up duration not specified for the sole survivor). Of those patients that received definitive intervention for TE (*n* = 25), 6/12 patients with embolectomy survived compared to 4/7 that underwent a lung resection and 4/6 who received chemotherapy alone.

## Discussion

### Clinical presentation

Individuals with TE have a range of clinical findings from asymptomatic to hemodynamic instability resulting in death [17]. Within this series, dyspnea was the most common clinical manifestation which is in line with other studies [7, 12, 18]. TE is often discovered after failure of anticoagulation for suspected VTE [14, 19, 20]. As with this case report herein, TE in sarcomas commonly present in an acute manner whereas, TE in non-sarcoma settings are predominantly subacute [14, 20].

### Pathophysiology

TE can develop from several mechanisms. Pulmonary artery embolism may occur but lymphatic invasion with mediastinal and pulmonary lymphatic extension is more common [21]. Diffuse tumor within the pulmonary perivascular lymphatics is termed carcinomatosis lymphangitis and may lead to compression of alveoli and capillaries (with or without arterial invasion) [21]. These mechanisms are not mutually exclusive, as TE can occur from several mechanisms concurrently [22]. However, pulmonary endarteritis is the common end result [22]. Similar mechanisms are responsible for TE in sarcoma [22, 23].

The risks factors underlying TE development are not well characterized [14]. This series revealed leiomyosarcomas and chondrosarcomas are the most frequently reported STS and bone sarcomas with TE, respectively. It is unclear whether this propensity is related to reporting biases or inherent biological characteristics of these histologies. Additionally, location may be important for TE development. STS are most commonly located in the lower extremity; yet, increased TE publications derived from the retroperitoneum were found.

### Sequelae of tumor embolism: pulmonary hypertension and cor pulmonale

Within the pulmonary artery, TE may resolve, remain latent, or progress [22]. Progression can lead to increased pulmonary vascular resistance and pulmonary hypertension. Chronic findings of pulmonary hypertension include intimal fibrosis and extension of smooth muscle into the distal pulmonary arterial tree [7]. A subset of patients with pulmonary hypertension will progress to cor pulmonale through increased afterload on the right heart.

Similar to other studies, evidence of cor pulmonale was present in less than 50% of cases within this series [24]. Most patients who develop neoplastic pulmonary hypertension will die from its sequelae in an average of 4 months from the onset of symptoms [7]. Overall mortality in this series was 64.3%; however, the mortality rate climbed to 88.9% among those who were also diagnosed with cor pulmonale.

### Diagnostic evaluation

The diagnosis of TE is challenging and may not be established until post-mortem evaluation as evidenced by 22.2% of cases within this series. CT is considered the gold standard for the diagnosis of TE [17, 25]. A beaded appearance of the pulmonary vasculature, low attenuation filling defects, and progressive dilation of the pulmonary artery may help to distinguish VTE from TE [17, 24, 26, 27]. Other diagnostic modalities have also been suggested including ventilation perfusion scan, bone scintigraphy, angiogram, echocardiogram, and histologic verification using endoscopic bronchial ultrasound and pulmonary artery catheter aspiration [7, 9, 10, 12, 17, 20, 26–28].

### Management

TE is frequently mistaken for VTE and anticoagulation is improperly initiated. Anticoagulation and thrombolysis are not useful for TE and considered an absolute contraindication as it may result in hemoptysis [12, 19, 29, 30]. In this series, 21 patients received anticoagulation while 3 patients also received thrombolysis. Surgical embolectomy was the most commonly utilized therapeutic strategy in this series without the absolute requirement for cardiopulmonary bypass [29, 31]. Additional measures for TE management involve pulmonary lobectomy or pneumonectomy [17]. Chemosensitive tumors may benefit from chemotherapy and have been considered by some groups to be the mainstay of treatment [24, 32]. When pulmonary embolectomy is not feasible or contraindicated, additional measures should be considered. Successful application of AngioJet has been reported for TE in a patient with renal cell carcinoma but remains experimental for sarcoma [33]. Additionally, IVC filters have been utilized for subdiaphgramtic tumors [12]. Notably, intraoperatively cardiovascular collapse during evacuation of a sarcoma TE has been previously described and highlights the potentially tenuous hemodynamic state [20, 34].

Limitations of this study include the limited reports of sarcoma TE. The rarity of TE in sarcoma and underappreciation of TE as a source of PE have led to misdiagnoses. Additionally, several cases did not have sufficient information to make comparisons across all categories that were assessed. Several reports were carried out before the advent and widespread availability of newer technologies (e.g., PET scan). Thus, their merit may not be fully appreciated and the rarity of this phenomenon in conjunction with a multitude of histologic sarcoma subtypes makes research investigations challenging.

## Conclusion

Sarcomas have an increased propensity for the development of emboli [35]. TE account for a minority of PE in sarcomas but are associated with a high mortality rate especially, when cor pulmonale has resulted. A high index of suspicion should be present in individuals with sarcomas who also present with respiratory-related symptoms, particularly dyspnea. While data is limited, a CT chest and echocardiogram may be a reasonable non-invasive approach to evaluate for TE and assess for sequela of PE while minimizing false positive results. Pulmonary embolectomy is the most common definitive approach for TE. Together, this information highlights the collective experience with this rare but potentially lethal complication of STS and bone sarcomas.

## Additional files

Additional file 1: Clinical data: Presentation and diagnosis. Clinical presentation and diagnosis of cases containing tumor embolism secondary to soft tissue and bone sarcoma. (DOCX 32 kb)

Additional file 2: Clinical data: Venous extension and management. Presence of local venous tumor extension and clinical management of cases containing tumor embolism secondary to soft tissue and bone sarcoma. (DOCX 25 kb)

## Abbreviations

PE: pulmonary embolism
STS: soft tissue sarcoma
VTE: venous thromboembolism
