# Supplementary material for: Pulmonary tumor embolism secondary to soft tissue and bone sarcomas: a case report and literature review
Source: World J Surg Oncol. 2017 Aug 30;15:168. doi: 10.1186/s12957-017-1223-3 (PMC5577830; doi:10.1186/s12957-017-1223-3)
Supplement: Supplementary file 1 — Clinical data: Presentation and diagnosis. Clinical presentation and diagnosis of cases containing tumor embolism secondary to soft tissue and bone sarcoma. (DOCX 32 kb) [file 12957_2017_1223_MOESM1_ESM.docx]

| **Case**  **Additional File 1.** Clinical data: presentation and diagnosis. | **Study** | **Age** | **Sex** | **Histology** | **Location** | **Pulmonary Symptoms** | **Onset** | **Antemortem Diagnosis** | **Diagnostic Evaluation** | **Pulmonary HTN** | **Cor pulmonale** |
| --- | --- | --- | --- | --- | --- | --- | --- | --- | --- | --- | --- |
| 1 | Habino *et al.* | 62 | F | Carcinosarcoma | Uterine | cough | NS | yes | CT x 3 | yes | yes |
|  |  |  |  |  |  | hemoptysis |  |  | TTE |  |  |
|  |  |  |  |  |  |  |  |  | PET |  |  |
|  |  |  |  |  |  |  |  |  | Bronchoscopy |  |  |
|  |  |  |  |  |  |  |  |  | V/Q |  |  |
|  |  |  |  |  |  |  |  |  |  |  |  |
| 2 | Hayashida *et al.* | 25 | F | Chondrosarcoma | Lower extremity | dyspnea | acute | Yes | CXR | yes | yes |
|  |  |  |  |  |  |  |  |  | V/Q |  |  |
|  |  |  |  |  |  |  |  |  | CT |  |  |
|  |  |  |  |  |  |  |  |  | TTE |  |  |
|  |  |  |  |  |  |  |  |  | Angiogram |  |  |
|  |  |  |  |  |  |  |  |  |  |  |  |
| 3 | Morgan *et al.* | 18 | M | Chrondrosarcoma | Pelvis | dyspnea | acute | yes | CT | yes | yes |
|  |  |  |  |  |  |  |  |  | TEE |  |  |
|  |  |  |  |  |  |  |  |  |  |  |  |
|  |  |  |  |  |  |  |  |  |  |  |  |
| 4 | Newkirk *et al.* | 32 | F | Chondrosarcoma | Pelvis | dyspnea | subacute | yes | CT | yes | yes |
|  |  |  |  |  |  | chest pain |  |  | TEE |  |  |
|  |  |  |  |  |  |  |  |  |  |  |  |
| 5 | Pinder *et al.* | 9 | F | Chondrosarcoma | Upper Extremity | cough | subacute | yes | CXR | yes | yes |
|  |  |  |  |  |  |  |  |  | CT |  |  |
|  |  |  |  |  |  |  |  |  | TTE |  |  |
|  |  |  |  |  |  |  |  |  | TEE |  |  |
|  |  |  |  |  |  |  |  |  |  |  |  |
| 6 | Abdulaziz *et al.* | 30 | M | Pleomorphic sarcoma | Upper Extremity | none- intubated | acute | no | CXR | NS | yes |
|  |  |  |  |  |  |  |  |  | TEE |  |  |
|  |  |  |  |  |  |  |  |  |  |  |  |
| 7 | Ahmed *et al.* | 65 | F | Osteosarcoma | Lower extremity | dyspnea | acute | no | CXR | NS | NS |
|  |  |  |  |  |  |  |  |  | V/Q |  |  |
|  |  |  |  |  |  |  |  |  |  |  |  |
| 8 | Laurain *et al.* | 13 | F | Osteosarcoma | Upper Extremity | dyspnea | acute | no | CXR | NS | NS |
|  |  |  |  |  |  |  |  |  |  |  |  |
|  |  |  |  |  |  |  |  |  |  |  |  |
| 9 | Chamorrow *et al.* | 51 | F | Synovial cell sarcoma | Retroperitoneum | dyspnea | subacute | yet | CTx2 | No | No |
|  |  |  |  |  |  |  |  |  | PET |  |  |
|  |  |  |  |  |  |  |  |  | TTE |  |  |
|  |  |  |  |  |  |  |  |  | EBUS-FNA |  |  |
|  |  |  |  |  |  |  |  |  |  |  |  |
| 10 | Chandrasekharan *et al.* | 19 | M | Chondrosarcoma | Pelvis | dyspnea | acute | yes | CXR | NS | yes |
|  |  |  |  |  |  |  |  |  | CT |  |  |
|  |  |  |  |  |  |  |  |  | Echo |  |  |
|  |  |  |  |  |  |  |  |  |  |  |  |
| 11 | Grab *et al.* | 45 | F | Pleomorphic sarcoma | Thyroid | dyspnea | subacute | no | CXR | NS | NS |
|  |  |  |  |  |  | chest pain |  |  | CT |  |  |
|  |  |  |  |  |  |  |  |  |  |  |  |
|  |  |  |  |  |  |  |  |  |  |  |  |
|  |  |  |  |  |  |  |  |  |  |  |  |
| 12 | Wakasaka *et al.* | 16 | F | Osteosarcoma | Lower Extremity | cough | subabcute | no | CXR | NS | NS |
|  | Patient 1 |  |  |  |  | hemoptysis |  |  |  |  |  |
|  |  |  |  |  |  | dyspnea |  |  |  |  |  |
|  |  |  |  |  |  |  |  |  |  |  |  |
| 13 | Wakasaka *et al.* | 16 | F | Osteosarcoma | Lower Extremity | dyspnea | acute | no | none | NS | NS |
|  | Patient 2 |  |  |  |  |  |  |  |  |  |  |
|  |  |  |  |  |  |  |  |  |  |  |  |
| 14 | Yutani *et al.* | 25 | F | Chrondrosarcoma | Lower Extremity | dyspnea | NS | yes | V/Q | yes | yes |
|  |  |  |  |  |  | cough |  |  | Angiogram |  |  |
|  |  |  |  |  |  |  |  |  |  |  |  |
|  |  |  |  |  |  |  |  |  |  |  |  |
| 15 | Shepard *et al.* | 50 | M | Chondrosarcoma | Pelvis | NS | NS | yes | CXR | NS | NS |
|  | Patient 1 |  |  |  |  |  |  |  | CT |  |  |
|  |  |  |  |  |  |  |  |  | Angiogram |  |  |
|  |  |  |  |  |  |  |  |  | Bone scan |  |  |
|  |  |  |  |  |  |  |  |  |  |  |  |
| 16 | Shepard *et al.* | 18 | M | Osteosarcoma | Pelvis | NS | NS |  | CXR | NS | NS |
|  | Patient 2 |  |  |  |  |  |  |  | CT |  |  |
|  |  |  |  |  |  |  |  |  | Angiogram |  |  |
|  |  |  |  |  |  |  |  |  |  |  |  |
| 17 | Benditt *et al.* | 55 | F | Chondrosarcoma | Pelvis | dyspnea | subacute | yes | CXR | NS | NS |
|  |  |  |  |  |  | chest pain |  |  | CT |  |  |
|  |  |  |  |  |  | cough |  |  |  |  |  |
|  |  |  |  |  |  |  |  |  |  |  |  |
| 18 | Leung *et al.* | 22 | F | Chondrosarcoma | Pelvis | NS | acute | yes | V/Q | NS | NS |
|  |  |  |  |  |  |  |  |  | TTE |  |  |
|  |  |  |  |  |  |  |  |  | TEE |  |  |
|  |  |  |  |  |  |  |  |  |  |  |  |
| 19 | Schwartz *et al.* | 70 | M | Chondrosarcoma | Rib | dyspnea | NS | yes | CXR x2 | yes | NS |
|  |  |  |  |  |  |  |  |  | V/Q x3 |  |  |
|  |  |  |  |  |  |  |  |  | Angiogram |  |  |
|  |  |  |  |  |  |  |  |  |  |  |  |
|  |  |  |  |  |  |  |  |  |  |  |  |
| 20 | McDonald *et al.* | 48 | F | Leiomyosarcoma | Uterus | dyspnea | NS | yes | CT | NS | NS |
|  |  |  |  |  |  |  |  |  | Tumor biopsy |  |  |
|  |  |  |  |  |  |  |  |  |  |  |  |
| 21 | Gentle *et al.* | 73 | F | Leiomyosarcoma | Lower Extremity | dyspnea | subabute | yes | CXR | yes | yes |
|  |  |  |  |  |  |  |  |  | V/Q |  |  |
|  |  |  |  |  |  |  |  |  | Angiogram |  |  |
|  |  |  |  |  |  |  |  |  |  |  |  |
| 22 | Demoulin *et al.* | 53 | F | Leiomyosarcoma | Retroperitoneum | dyspnea | NS | yes | CXR | NS | yes |
|  |  |  |  |  |  |  |  |  | Angiogram |  |  |
|  |  |  |  |  |  |  |  |  | V/Q |  |  |
|  |  |  |  |  |  |  |  |  |  |  |  |
| 23 | Arbeit *et al.* | 52 | F | Leiomyosarcoma | Retroperitoneum | cough | NS | yes | Bronchoscopy | NS | NS |
|  |  |  |  |  |  | hemoptysis |  |  | CXR |  |  |
|  |  |  |  |  |  |  |  |  |  |  |  |
|  |  |  |  |  |  |  |  |  |  |  |  |
| 24 | Castleman *et al.* | 62 | M | Chondrosarcoma | Rib | dyspnea | subacute | no | CXR x 2 | NS | yes |
|  |  |  |  |  |  | cough |  |  | Sputum cytology |  |  |
|  |  |  |  |  |  | hemoptysis |  |  |  |  |  |
|  |  |  |  |  |  |  |  |  |  |  |  |
| 25 | Kruger *et al.* | 49 | M | Leiomyosarcoma | Retroperitoneum | dyspnea | NS | yes | NS | NS | NS |
|  |  |  |  |  |  |  |  |  |  |  |  |
| 26 | Schmid *et al.* | 52 | M | Synovial sarcoma | Retroperitoneum | NS | NS | yes | CTx3 | NS | NS |
|  |  |  |  |  |  |  |  |  | EBUS-FNA |  |  |
|  |  |  |  |  |  |  |  |  |  |  |  |
| 27 | Dua *et al.* | 52 | M | Undifferentiated | Liver | dyspnea | acute | yes | TTE x2 | yes | no |
|  |  |  |  |  |  |  |  |  | CTx3 |  |  |
|  |  |  |  |  |  |  |  |  |  |  |  |
| 28 | Hahn *et al.* | 22 | F | Osteosaroma | Pelvis | dyspnea | acute | yes | CXR | yes | yes |
|  |  |  |  |  |  |  |  |  | V/Q |  |  |
|  |  |  |  |  |  |  |  |  | Angiogram |  |  |
|  |  |  |  |  |  |  |  |  |  |  |  |
|  |  |  |  |  |  |  |  |  |  |  |  |
| 29 | Hayashida *et al.* | 25 | F | Chrondrosarcoma | Lower Extremity | dyspnea | chronic | yes | CXR | yes | yes |
|  |  |  |  |  |  |  |  |  | Bone scan |  |  |
|  |  |  |  |  |  |  |  |  | V/Q |  |  |
|  |  |  |  |  |  |  |  |  | CT |  |  |
|  |  |  |  |  |  |  |  |  | Angiogram |  |  |
|  |  |  |  |  |  |  |  |  |  |  |  |
| 30 | Latchana *et al.* | 36 | F | Myxofibrosarcoma | Upper Extremity | dyspnea | acute | yes | CXR | yes | yes |
|  |  |  |  |  |  |  |  |  | CT |  |  |
|  |  |  |  |  |  |  |  |  | TTE |  |  |
|  |  |  |  |  |  |  |  |  |  |  |  |
| 31 | Budiri *et al.* | 48 | M | Osteosarcoma | Lower Extremity | dyspnea | NS | yes | CT | NS | NS |
|  |  |  |  |  |  |  |  |  |  |  |  |
|  |  |  |  |  |  |  |  |  |  |  |  |
| 32 | Garcia-Covarrubias *et al.* | 66 | M | Rhabdosarcoma | Retroperitoneum | dyspnea | acute | yes | CT | NS | NS |
|  |  |  |  |  |  |  |  |  | MRI |  |  |
|  |  |  |  |  |  |  |  |  | TTE |  |  |
|  |  |  |  |  |  |  |  |  | TEE |  |  |
|  |  |  |  |  |  |  |  |  |  |  |  |
| 33 | Lalueza *et al.* | 20 | M | osteosarcoma | Lower Extremity | dyspnea | acute | yes | CT | NS | NS |
|  |  |  |  |  |  |  |  |  |  |  |  |
| 34 | Peixoto *et al.* | 45 | M | chondrosarcoma | Lower Extremity | asympatomatic | NS | yes | CT | NS | NS |
|  |  |  |  |  |  |  |  |  | Lung Biopsy |  |  |
|  |  |  |  |  |  |  |  |  |  |  |  |
| 35 | Shao *et al.* | 15 | M | Osteosarcoma | Lower Extremity | dyspnea | acute | yes | CT x 2 | yes | yes |
|  |  |  |  |  |  | hemoptysis |  |  |  |  |  |
|  |  |  |  |  |  |  |  |  |  |  |  |
|  |  |  |  |  |  |  |  |  |  |  |  |
| 36 | Shapario *et al.* | 17 | M | Osteosarcoma | Lower Extremity | dyspnea | acute | yes | CTx 3 | NS | NS |
|  |  |  |  |  |  | chest pain |  |  | PET |  |  |
|  |  |  |  |  |  | hemoptysis |  |  | TTE |  |  |
|  |  |  |  |  |  |  |  |  |  |  |  |
| 37 | Ting *et al.* | 38 | F | Chondrosarcoma | Scapula | dyspnea | NS | yes | CXR | NS | NS |
|  |  |  |  |  |  | chest pain |  |  | CT |  |  |
|  |  |  |  |  |  |  |  |  | Spirometry |  |  |
|  |  |  |  |  |  |  |  |  |  |  |  |
| 38 | Ting *et al.* | 29 | M | Chondrosarcoma | Lower Extremity | dyspnea | NS | yes | CTx 3 | NS | yes |
|  |  |  |  |  |  | cough |  |  | Bronchoscopy |  |  |
|  |  |  |  |  |  | chest pain |  |  |  |  |  |
|  |  |  |  |  |  |  |  |  |  |  |  |
| 39 | Ting *et al.* | 18 | F | Osteosarcoma | Lower Extremity | asymptomatic | NS | yes | CT | NS | NS |
|  |  |  |  |  |  |  |  |  |  |  |  |
|  |  |  |  |  |  |  |  |  |  |  |  |
| 40 | Soares *et al.* | 10 | M | Rhabdomyosarcoma | Retroperitoneum | dyspnea | NS | no | CXR | NS | yes |
|  | Patient 1 |  |  |  |  |  |  |  |  |  |  |
|  |  |  |  |  |  |  |  |  |  |  |  |
| 41 | Soares *et al.* | 12 | F | Chondrosarcoma | Pelvis | dyspnea | acute | no | CXR | NS | yes |
|  | Patient 2 |  |  |  |  |  |  |  |  |  |  |
|  |  |  |  |  |  |  |  |  |  |  |  |
| 42 | Schwarz *et al.* | 70 | M | Chondrosarcoma | Rib | dyspnea | NS | yes | CXR | yes | no |
|  |  |  |  |  |  | chest pain |  |  | V/Q x2 |  |  |
|  |  |  |  |  |  |  |  |  | Angiogram |  |  |
|  |  |  |  |  |  |  |  |  |  |  |  |
| 43 | Booth *et al.* | 9 | F | Osteosarcoma | Lower Extremity | cough | NS | yes | CXR | NS | NS |
|  |  |  |  |  |  | hemoptysis |  |  | Lung Biopsy |  |  |
|  |  |  |  |  |  |  |  |  | V/Q |  |  |
|  |  |  |  |  |  |  |  |  |  |  |  |
| 44 | Hoefnagel *et al.* | 47 | M | Osteosarcoma | Lower Extremity | cough | NS | yes | CXR | NS | NS |
|  |  |  |  |  |  | dyspnea |  |  | CT |  |  |
|  |  |  |  |  |  |  |  |  | V/Q x 3 |  |  |
|  |  |  |  |  |  |  |  |  | Bone Scintigraphy x 2 |  |  |
|  |  |  |  |  |  |  |  |  |  |  |  |
| 45 | Rastogi *et al.* | - | - | Osteosarcoma | Lower Extremity | dyspnea | acute | no | none | NS | yes |

Clinical presentation and diagnosis of cases containing tumor embolism secondary to soft tissue and bone sarcoma. Abbreviations: CXR: chest x-ray; CT: computed tomography; TTE: transthoracic echocardiogram; TEE: transesophageal echocardiography; V/Q: ventilation/perfusion scan; EBUS-FNA: endobronchial ultrasound guided fine needle aspiration; NS: not specified.
