# Supplementary material for: Pulmonary tumor embolism secondary to soft tissue and bone sarcomas: a case report and literature review
Source: World J Surg Oncol. 2017 Aug 30;15:168. doi: 10.1186/s12957-017-1223-3 (PMC5577830; doi:10.1186/s12957-017-1223-3)
Supplement: Supplementary file 2 — Clinical data: Venous extension and management. Presence of local venous tumor extension and clinical management of cases containing tumor embolism secondary to soft tissue and bone sarcoma. (DOCX 25 kb) [file 12957_2017_1223_MOESM2_ESM.docx]

**Additional File 2.** Clinical data: Venous extension and management

| **Case** | **Study** | **Local Venous Extension** | **Anticoagulation** | **Definitive TE Treatment** | **Management of primary tumor** | **Death (time)** |
| --- | --- | --- | --- | --- | --- | --- |
| 1 | Habino *et al.* | no | yes | lobectomy | resection | yes (12 months) |
|  |  |  |  |  |  |  |
| 2 | Hayashida *et al.* | no | Yes | embolectomy (failed) | resection | yes (19 days) |
|  |  |  |  |  | chemotherapy |  |
|  |  |  |  |  |  |  |
| 3 | Morgan *et al.* | no | Yes | embolectomy (failed) | None | yes (5 days) |
|  |  |  |  |  |  |  |
| 4 | Newkirk *et al.* | yes | yes | embolectomy | chemotherapy | yes ( 2 months) |
|  |  |  |  |  | resection |  |
|  |  |  |  |  |  |  |
| 5 | Pinder *et al.* | no | yes | embolectomy | chemotherapy | no (NS) |
|  |  |  |  |  |  |  |
| 6 | Abdulaziz *et al.* | no | no | embolectomy | NS | yes (3 months) |
|  |  |  |  |  |  |  |
| 7 | Ahmed *et al.* | no | yes | none | resection | yes (30 hours) |
|  |  |  |  |  |  |  |
| 8 | Laurain *et al.* | no | no | none | resection | yes (2 days) |
|  |  |  |  |  |  |  |
| 9 | Chamorrow *et al.* | no | yes | chemotherapy | resection | no (alive at 9 months) |
|  |  |  |  |  | chemotherapy |  |
|  |  |  |  |  | radiation |  |
|  |  |  |  |  |  |  |
| 10 | Chandrasekharan *et al.* | yes | yes | embolectomy | chemotherapy | no (NS) |
|  |  |  |  |  |  |  |
| 11 | Grab *et al.* | yes | no | none | none | yes (10 days) |
|  |  |  |  |  |  |  |
| 12 | Wakasaka *et al.* | no | no | none | resection | yes (1 month) |
|  |  |  |  |  | chemotherapy |  |
|  |  |  |  |  |  |  |
| 13 | Wakasaka *et al.* | no | no | none | chemotherapy | yes (4 weeks) |
|  |  |  |  |  |  |  |
|  |  |  |  |  |  |  |
| 14 | Yutani *et al.* | no | no | embolectomy | resection | yes (10 days) |
|  |  |  |  |  | radiation |  |
|  |  |  |  |  |  |  |
| 15 | Shepard *et al.* | no | NS | pulmonary wedge resection | resection | NS |
|  |  |  |  |  |  |  |
| 16 | Shepard *et al.* | no | NS | NS | resection | NS |
|  |  |  |  |  |  |  |
| 17 | Benditt *et al.* | no | no | none | NS | yes (1 month) |
|  |  |  |  |  |  |  |
| 18 | Leung *et al.* | no | yes | embolectomy | chemotherapy | no (alive at 4 months) |
|  |  |  |  |  | radiation |  |
| 19 | Schwartz *et al.* | no | yes | none | resection | yes (6 months) |
|  |  |  |  |  |  |  |
| 20 | McDonald *et al.* | yes | no | none | resection | no (NS) |
|  |  |  |  |  |  |  |
| 21 | Gentle *et al.* | no | yes | none | resection | yes (5 weeks) |
|  |  |  |  |  |  |  |
| 22 | Demoulin *et al.* | no | no | embolectomy | NS | yes (intra-operatively) |
|  |  |  |  |  |  |  |
| 23 | Arbeit *et al.* | yes | no | pneumopnectomy | resection | yes (4 months) |
|  |  |  |  |  | chemotherapy |  |
|  |  |  |  |  | radiation |  |
|  |  |  |  |  |  |  |
| 24 | Castleman *et al.* | no | no | none | resection | yes (11 days) |
|  |  |  |  |  |  |  |
| 25 | Kruger *et al.* | no | no | embolectomy | resection | no (alive 6 months) |
|  |  |  |  |  |  |  |
| 26 | Schmid *et al.* | no | yes | lobectomy | resection | no (NS) |
|  |  |  |  |  | chemotherapy |  |
|  |  |  |  |  |  |  |
| 27 | Dua *et al.* | no | yes | embolectomy | resection | no (NS) |
|  |  |  |  |  | chemotherapy |  |
|  |  |  |  |  |  |  |
| 28 | Hahn *et al.* | no | yes | none | chemoradiatinon | yes (8 hours) |
|  |  |  |  |  |  |  |
|  |  |  |  |  |  |  |
| 29 | Hayashida *et al.* | no | yes | none | chemotherapy | yes (19 days) |
|  |  |  |  |  | resection |  |
|  |  |  |  |  |  |  |
| 30 | Latchana *et al.* | yes | yes | none | resection | yes (1 day) |
|  |  |  |  |  | chemotherapy |  |
|  |  |  |  |  | radiation |  |
|  |  |  |  |  |  |  |
| 31 | Budiri *et al.* | no | no | chemotherapy | chemotherapy | no (alive at 6 weeks) |
|  |  |  |  | pneumonectomy | resection |  |
|  |  |  |  |  |  |  |
| 32 | Garcia-Covarrubias *et al.* | yes | no | embolectomy | resection | no (alive at 13 days) |
|  |  |  |  |  |  |  |
| 33 | Lalueza *et al.* | no | no | none | none | yes (NS) |
|  |  |  |  |  |  |  |
| 34 | Peixoto *et al.* | no | no | chemotherapy | resection | no (NS) |
|  |  |  |  |  |  |  |
|  |  |  |  |  |  |  |
| 35 | Shao *et al.* | no | yes | none | resection | yes (8 weeks) |
|  |  |  |  |  |  |  |
| 36 | Shapario *et al.* | no | yes | pneumopnectomy | resection | no (alive at 29 months) |
|  |  |  |  |  | chemotherapy |  |
|  |  |  |  |  |  |  |
| 37 | Ting *et al.* | no | no | lung resection | radiation | no (alive at 3 years) |
|  |  |  |  | chemotherapy | chemotherapy |  |
| 38 | Ting *et al.* | no | no | chemotherapy | amputation | NS |
|  |  |  |  |  |  |  |
| 39 | Ting *et al.* | no | no | chemotherapy | chemorads | no (alive at 6 months) |
|  |  |  |  |  |  |  |
| 40 | Soares *et al.* | no | no | chemotherapy | chemotherapy | yes (1 month) |
|  | Patient 1 |  |  |  |  |  |
| 41 | Soares *et al.* | no | no | none | resection | yes (45 days) |
|  | Patient 2 |  |  |  | chemotherapy |  |
|  |  |  |  |  |  |  |
| 42 | Schwarz *et al.* | no | yes | none | resection | yes (7months) |
|  |  |  |  |  |  |  |
| 43 | Booth *et al.* | yes | yes | none | resection | yes (1 week) |
|  |  |  |  |  | chemotherapy |  |
|  |  |  |  |  |  |  |
| 44 | Hoefnagel *et al.* | no | yes | chemotherapy | resection | no (lost to follow up) |
|  |  |  |  |  | radiation |  |
|  |  |  |  |  |  |  |
| 45 | Rastogi *et al.* | no | no | none | resection | yes (3 hours) |

Presence of local venous tumor extension and clinical management of cases containing tumor embolism secondary to soft tissue and bone sarcoma. Abbreviations: NS: not specified.
